# COVID 19 meets changing traditional care systems for the elderly and a budding social work practice. Reflections for geriatric care in Ghana

**DOI:** 10.1177/1473325020973323

**Published:** 2021-03

**Authors:** Charles Selorm Deku, John Boulard Forkuor, Eric Agyemang

**Affiliations:** Department of Sociology and Social Work, Faculty of Social Sciences, 98763 Kwame Nkrumah University of Science and Technology (KNUST), Kumasi, Ghana

**Keywords:** Older adults, family caregiving, social work practice, COVID-19, elderly care, extended family

## Abstract

Starting in December 2019 in Wuhan China, the novel coronavirus (COVID 19) disease has reached 216 countries with 6, 140, 934 confirmed cases and 373, 548 deaths as at 2nd June, 2020 globally Ghana, with an estimated population of 31,014,508 has recorded 8, 297 confirmed cases, 2, 986 recoveries and 38 deaths with 5, 273 active cases as at the same date. All but one of the 16 administrative regions have recorded confirmed cases with the highest case numbers in the more urban regions of the country. Considering that one of the highest risk populations in the wake of the coronavirus outbreak is the elderly population, this brief essay examines the state of elderly care in Ghana in relation to this pandemic. The paper reflects on the state of care needs for the elderly, current elderly care systems, inadequacy of data on elderly population and social work practice in Ghana. It also raises questions on the preparedness of current elderly care systems and general social work practice in Ghana amidst COVID 19. The paper recommends professionalization of geriatric care and formalization of community-based care for the elderly in Ghana as the way forward.

## Systems of care for elderly care in Ghana: How relevant is extended family care today?

The role of kin and kith in care for the elderly in Ghana cannot be overlooked but its complexities do not submit to a simple summary. People aged 60+ in Ghana constitute 7.2% of the general population and this is estimated to rise from 1.5 million in 2010 to 2.8 million by 2030 (Mba, 2010). I see even a greater growth in these numbers in the coming years because of some general successes in healthcare and economic development in the country. In one of the earliest studies on elderly care in Ghana, Apt (1993) acknowledged that care for the elderly is influenced by socioeconomic conditions. Apt (1993) however noted the disintegrating social security (extended family) for the elderly even though some researchers still regard extended family support as a channel of delivering care to the elderly. Just some few years ago in my village and I believe in most rural close-knit communities in Ghana, hunger and lack of care for the elderly was difficult to find. Admittedly, supplying the needs of the aged falls first on their immediate family; but was the village not just one big family with almost everyone assuming responsibility for one another? I remember how different families from all ends of the village brought food, water and sometimes medication to one frail old man with neither wife nor child, who lived behind our window. It is reported he never lived responsibly but he still had some care from the other villagers. That was the norm in such communities – the needs of the elderly were the needs of the collective whole but the situation has however undergone a lot of change over time.

Respect and reciprocity are the central motivations for elderly care in rural Ghanaian communities if not the whole country. Children are regarded as the insurance for care and support of the elderly. Thus, those with no child or who have not lived responsibly faced more challenges in meeting their care needs sustainably. The suggestion that old age confers respect was contested by Van der Geest (2002) who identified that old age and a respectable status are not always a given since there are old, miserable, poor and hungry people. Ofori-Dua (2014) emphasized the vulnerability of the elderly by explaining that because of poor economic situations and difficulties in saving against the future, low education and the resulting challenge in accessing social security interventions, old age and poverty are associated especially in rural Ghana. In the urban areas of the country where the young survive on their own and largely earn better than the aged, support for the aged based on respect and a sense of “indebtedness to the aged” is almost absent. More so, the aged living in urban Ghana without children face a greater tendency to have more unmet care needs. Destitution and ageing are almost synonymous from the foregoing. Apt (1993: 101) explained destitution as “a question of old, feeble minded, or sick people”. Old age does not only pose health threats and make the elderly more at risk but they also have several care-needs (Van der Geest, 2002).

Gradually, the general Ghanaian life is changing from being one in a simple subsistence agrarian dwelling to a more urbanized, industrialized and individualized arrangement with even higher aged proportion. The changes in the structure of society reveal to me several emerging care needs and fewer sources of care for the aged. While the able-bodied struggle to survive, I wonder the coping mechanisms available to the at-risk aged population in the face of a pandemic like the COVID 19. Even the strongest economies and systems are being challenged by the effects of this pandemic. How would the at-risk elderly population wallowing in poverty find refuge in the face of a pandemic like this? These questions deserve even greater consideration taking into account the wide range of care needs of the elderly matched against the inadequacy of available formalized support in a country like Ghana.

Care needs for the elderly in Ghana range from hygiene care activities such as disposing of urine and emptying chamber pot, washing of clothes and making water available for bathing. This according to Van der Geest (2002) is because old smelly people lose dignity in their communities. To ridicule families because their aged parents lacked proper care used to be an effective way of ensuring that the aged in rural Ghana, some few years ago, had the needed care. Observing the situation in urban Ghana however shows that the potency of public ridicule as a means of ensuring hygiene care for the aged is waning because of the growing individualization in carving out identities. Very important also is food, financial needs, need for company, keeping an eye on the elderly (in the case of those with peculiar mental challenges) and a befitting funeral in the event of death of the elderly person (Van der Geest, 2002) among others. Musing over the plight of the aged living in poor urban locations without potable water, necessary social support, I see people who cannot maintain the needed hygiene, eat a balanced diet or have the emotional support they need in these trying times. Poverty, the shift from the extended family to more nuclearized families is gradually breaking the shelter the aged need to cope well with the current pandemic. What happens in the future should another pandemic of this form or worst erupt? Are we readying to bury the aged before their time?

Like everywhere else, the elderly in Ghana have underlying medical conditions that make them more unlikely to survive the novel corona virus in case they contract it. Ayernor (2012: 18) noted peculiar diseases among the elderly in Ghana by identifying that “45% had oral health problems, 33% were hypertensive, 14% reported having arthritis; 7% had been diagnosed with diabetes, 6% had a cardiovascular condition and 4.9% were receiving treatment for stroke or had been diagnosed with stroke”. According to the CDC COVID 19 Response Team (2020: 1), “31% of cases, 45% of hospitalizations, 53% of ICU admissions, and 80% of deaths associated with COVID-19 were among adults aged ≥65 years with the highest percentage of severe outcomes among persons aged ≥85 years”. The high risks faced by the elderly are obvious and even worse because of the inadequacy of sophisticated care facilities for treating coronavirus patients in Ghana. The threats posed by hunger, loneliness from neglect by the state and other actors during the pandemic coupled with the prevailing medical needs makes the aged more helpless in the case of Ghana. Social protection interventions have rather unfortunately largely targeted the *kayayei* (head potters) but the elderly is left to fend for themselves and worry about the pandemic. If the highest risk population in a pandemic like this is the most ignored, the adequacy of government efforts in dealing with the pandemic requires a sure reconsideration.

Only the elderly person who has invested care in their children or other relatives during their hay days tend to receive care at old age (Van der Geest, 2002). What constitutes care may be evasive and ambiguous due to differences in contexts. Care from extended family to the elderly in Ghana is managed on a daily basis with several improvisations (Van der Geest, 2002). Ofori-Dua (2014) also added that receipt of care for elderly people depends on adequacy of their social network and their degree of connectedness to the environment. The question that begs consideration is how the aged living in urban areas and in situations that offer no reliable support network are negotiating their survival. Changes in family solidarity, shifts from the lineage to conjugal family arrangements, low regard for elderly wisdom, migration and “monetization of life” and absence of formally organized care for the elderly make receiving of care from the extended family unreliable (Van der Geest, 2002: 18) especially in urban Ghana where the confirmed cases of COVID 19 are rife. Again, the lack of adequate data on aged, their specific care needs and formal care in developing countries like Ghana is a challenge to providing the effective care needed to reduce elderly morbidity and increase their longevity (Lafortune and Balestat, 2007; Tollman et al., 2008). From the foregoing, care for elderly is seen generally to be reciprocal (Mba, 2010; Ofori-Dua, 2014; Van der Geest, 2002) and emerging from respect (Van der Geest, 2002). A cursory observation of life in urban Ghana however defeats the effectiveness of the above explanations for the nature of elderly care.

## Social work practice readiness and response to COVID 19 in Ghana

Professional social work aims to help societies work better to serve its people. Institutionalization of professional practice in Ghana is just budding, or rather weak. This means the many elderly people and other vulnerable groups who would have benefited from professional social work would need to find alternatives. The profession in Ghana faces peculiar challenges in negotiating professional principle and traditional methods in addressing social problems through family support and networks (Baffoe and Dako-Gyeke, 2013). The Department of Social Welfare (now Department of Social Development) is the main government organisation responsible for overseeing welfare of the vulnerable in Ghana. The lack of adequate resourcing for the department, lack of active involvement in national development dialogue and policy implementation limits the success of the Department of Social Development. Readiness of the country Ghana to care for the aged has always remained a question, and even becomes a bigger issue in the face a pandemic like the COVID 19.

The response and social work interventions during this pandemic can be referred to as speculative and fraught with targeting discrepancies. Direct involvement of the Social Development Department and what could be called professional social work was absent. Rather, the National Disaster Management Organisation (NADMO) was tasked to reach out to the vulnerable through a food distribution program. Unsurprisingly, NADMO lacked the needed data and specifications of their target. In what could be described as a survival of the fittest approach to sharing relief items, NADMO failed to incorporate the needs of the elderly in their interventions. The difficulty in reaching frail aged people while maintaining the needed social and physical distancing protocols becomes a challenge. One would have thought the help of professionals would be sought on how to make specific efforts to help this most vulnerable group in the wake of the coronavirus outbreak.

## Concluding thoughts

Considering the number of social work students who graduate from Ghana’s universities, I believe a joint effort of the Social Development Department, government, and Social Work educators in gerontology and geriatrics to professionalize geriatric practice in Ghana is the way forward. A professional body that focuses on the elderly would be able to properly address their needs more efficiently. The presence of professional geriatric social workers in Ghana would enable the generation of adequate data on the elderly for improved targeting in extending social security needs to the elderly beyond this pandemic. This would also help in the long run to close the gap on unmet care needs of the elderly in urban Ghana in a more sustainable way.

It is further presented that formalization of care for the aged be considered in the long run. Formalized care offers ready data and prevents the discrepancies faced in reaching the elderly in situations like these. The remaining traces of extended family support for the elderly can still be maintained while adopting formalized and sustainable care. This is important to create a community within or close to the communities where the elderly dwell. Community supported care homes would ensure that the elderly do not lose contact with their families and kith and any support that may accrue from such networks to compliment care provided by professional caregivers. The collaboration between the remains of traditional care systems and formalized care is essential in providing adequate, sustainable care even to the elderly while keeping them away from constant contact from larger community which are more mobile and pose risks of unknowingly infecting them with the coronavirus.
